# Surgical Approach to Redo Open Living Donor Nephrectomy via the Same Flank Incision: A Case Report

**DOI:** 10.1155/crit/1599252

**Published:** 2026-08-24

**Authors:** Hung Ngoc Pham, Can Van Truong, Tuan Kim Nguyen, Phuc Dai Hong Vo, Anh Van Quoc Nguyen, Viet Huu Quoc Phan, Tuan Minh Truong, Thang Vuong Hoang, Hieu Van Le, John Edmondson, Kaitlyn Maddra, Lance Hampton

**Affiliations:** ^1^ Department of Urology, Hue Central Hospital, Hue, Vietnam, bvtwhue.com.vn; ^2^ Department of Urology, Virginia Commonwealth University Health System, Richmond, Virginia, USA, vcuhealth.org

**Keywords:** anaphylaxis, kidney transplantation, living kidney donation, open donor nephrectomy, redo surgery, retroperitoneal adhesions

## Abstract

Living donor nephrectomy is performed with donor safety as the highest priority, and repeat donor nephrectomy through the original incision is rarely reported in transplantation literature. We present the case of a 33‐year‐old male living kidney donor and a 48‐year‐old female recipient who underwent scheduled kidney transplantation. The initial procedure was aborted before vascular ligation because the recipient developed intraoperative anaphylactic shock after anesthesia induction. Three weeks later, transplantation was reattempted using the same donor flank incision. Dense retroperitoneal adhesions were encountered, requiring posterior peritoneal dissection to safely expose the renal hilum and surrounding structures. The donor kidney was successfully harvested and transplanted without postoperative complications. This case demonstrates that repeat open donor nephrectomy via the original incision may be feasible with carefully selected patients with a cautious surgical plan and multidisciplinary perioperative management.

## 1. Introduction

Living donor kidney transplantation is the preferred renal replacement therapy for many patients with end‐stage kidney disease [1]. Because donor nephrectomy provides no direct medical benefit to the donor, perioperative management must prioritize donor safety while preserving graft quality [2, 3]. Rarely, unexpected recipient‐related events after donor kidney exposure may necessitate abortion of the transplant procedure, creating ethical and surgical challenges. To our knowledge, reports of repeat donor nephrectomy via the same incision are extremely limited [4]. We report a rare case of repeat open living donor nephrectomy performed through the same flank incision after aborting initial transplantation due to intraoperative recipient anaphylaxis.

## 2. Methods and Ethical Considerations

Clinical data were retrospectively collected from the electronic medical records of the donor and recipient involved in this transplantation procedure. The study was conducted in accordance with the ethical principles of the Declaration of Helsinki (1964). Written informed consent for publication of clinical details and images was obtained from both the donor and the recipient. The Institutional Review Board of Hue Central Hospital waived approval for this case report because no experimental intervention was performed.

## 3. Case Presentation

### 3.1. Patient Information

**Figure 1:**
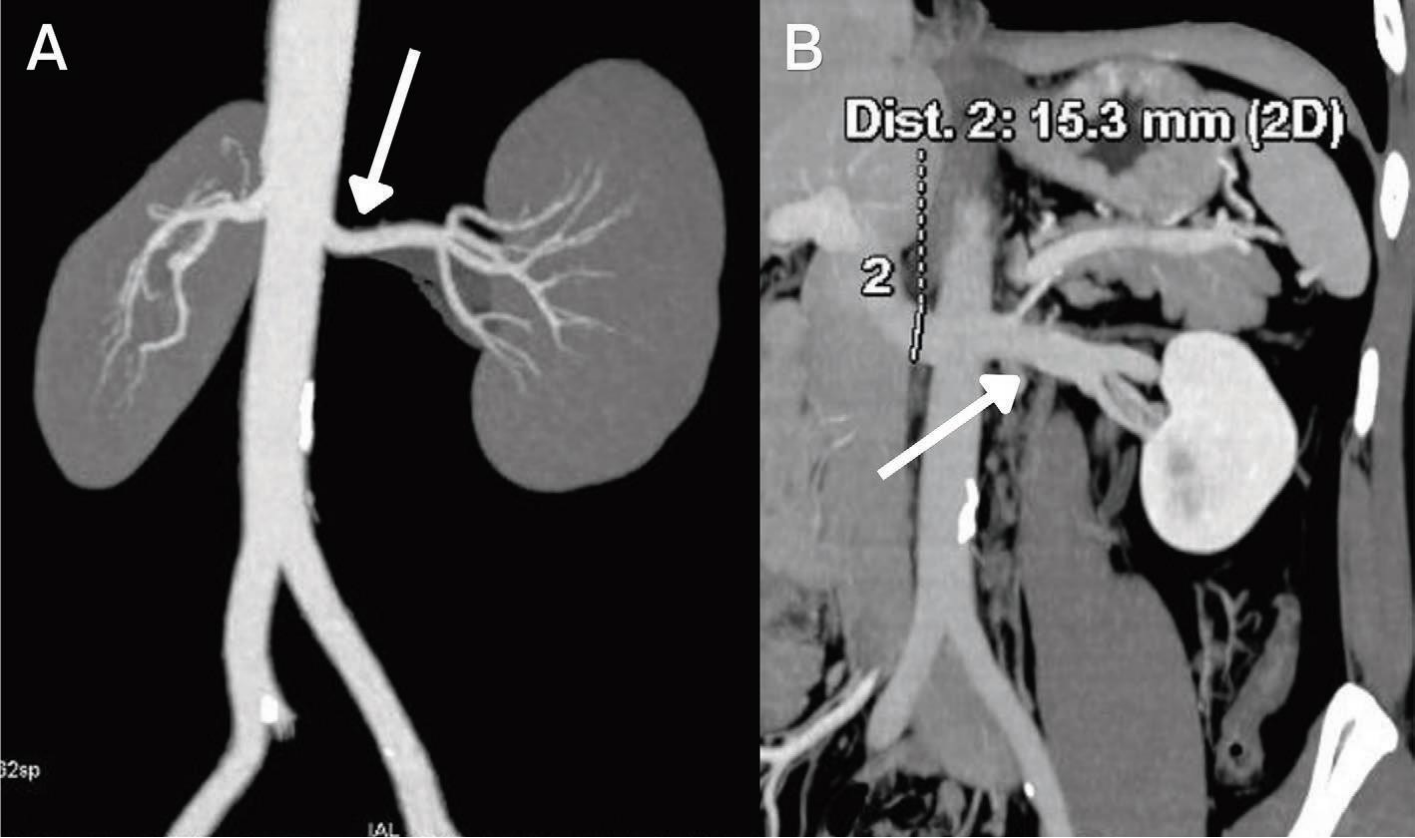
CTA imaging of donor kidney. (A) Left single renal artery (arrow). (B) Left single renal vein (arrow).

The living donor was a 33‐year‐old male with no significant medical history in the past. Preoperative computed tomography angiography (CTA) demonstrated bilateral single renal artery and single renal vein (Figure 1); the left kidney was selected for donation according to the institutional living donor protocol. The recipient was a 48‐year‐old female with end‐stage kidney disease secondary to acute glomerulonephritis and was receiving maintenance hemodialysis. She had a history of hypersensitivity reactions with weather and environment, especially presenting with urticaria and pruritus responsive to corticosteroids and antihistamines. Donor and recipient shared blood Group B Rh‐positive status. Human leukocyte antigen (HLA) testing showed matching at A11 and DRB1‐12, and pretransplant crossmatch and antibody screening were negative. The donor was a voluntary living unrelated kidney donor with no biological relationship to the recipient.

### 3.2. Preoperative Findings

Preoperative living donor evaluation revealed no medical contraindications to donation. The recipient′s immunologic profile was compatible with transplantation. Baseline renal function and other relevant perioperative variables are summarized in Table 1.

**Table 1:** Baseline characteristics, perioperative variables, and postoperative outcomes of the donor and recipient.

Variable	Donor	Recipient
Age/sex	33‐year‐old male	48‐year‐old female
Primary diagnosis	Healthy living donor	ESKD after acute glomerulonephritis
Blood group	B Rh‐positive	B Rh‐positive
Immunologic evaluation	Compatible crossmatch	Negative donor‐specific antibody
CT angiography	Single renal artery and vein bilaterally	No significant iliac vascular abnormality
Baseline creatinine/eGFR	Creatinine: 68.8 *μ*mol/L, eGFR: 66.5 mL/min/1.73 m^2^	Creatinine: 850 *μ*mol/L, eGFR: 5 mL/min/1.73 m^2^
Operative time	3 h	40 min after graft arrival
Estimated blood loss	200 mL	< 50 mL
Intraoperative fluids	1.5 L crystalloid	1 L crystalloid
Warm ischemia time	2 min	N/A
Cold ischemia time	N/A	20 min
Discharge creatinine/eGFR	Creatinine: 112 *μ*mol/L, eGFR: 52 mL/min/1.73 m^2^	Creatinine: 75 *μ*mol/L, eGFR: 84 mL/min/1.73 m^2^
3‐month follow‐up creatinine/eGFR	N/A	Creatinine: 72 *μ*mol/L, eGFR: 88 mL/min/1.73 m^2^
6‐month follow‐up creatinine/eGFR	N/A	Creatinine: 74 *μ*mol/L, eGFR: 85 mL/min/1.73 m^2^
12‐month follow‐up creatinine/eGFR	N/A	Creatinine: 71 *μ*mol/L, eGFR: 90 mL/min/1.73 m^2^
Complications	None reported	None reported
Postoperative hospital stay after reattempted transplantation	7 days	35 days

### 3.3. Donor Operation

The transplantation procedures were performed at Hue Central Hospital, Vietnam, in March 2025. After induction of general anesthesia and endotracheal intubation, a Foley catheter was placed, and the donor was positioned in the left lateral decubitus position. A 12‐cm left flank incision was made at the 11th–12th intercostal space. The abdominal wall muscles were dissected to approach the retroperitoneal space. Gerota fascia was opened, perirenal fat was mobilized, the ureter was dissected toward the iliac vessels, and the left renal artery and vein were exposed. The kidney had not been removed, and the renal vessels had not been divided when the recipient event suddenly occurred. After the transplant surgery was postponed, a drainage tube was placed, and the donor incision was closed in multiple layers, with the muscle layer closed using interrupted 1‐0 Vicryl sutures, whereas the subcutaneous tissue and skin were approximated using 3‐0 nylon sutures. The donor was extubated and transferred to the postoperative recovery unit for monitoring.

### 3.4. Recipient Event

The recipient was anesthetized and positioned supine for transplant approach. A Foley catheter was inserted. Approximately 3 min after administration of a muscle relaxant, she developed tachycardia exceeding 100 beats/min, hypotension to 60/40 mmHg, a weak pulse, and cyanosis, consistent with anaphylactic shock. She was treated according to anaphylaxis protocol, including high‐flow oxygen, isotonic intravenous fluids, adrenaline, intravenous methylprednisolone, and antihistamine therapy [5, 6]. Her vital signs and peripheral perfusion gradually improved after shock management. Given that recipient safety was the immediate priority and the donor kidney had not been removed, the transplant was postponed. The recipient was extubated and transferred to the intensive care unit for strict observation.

### 3.5. Multidisciplinary Reassessment and Timing

A multidisciplinary transplant team including urologists, nephrologists, anesthetists, and allergists reviewed the recipient‐related shock phenomenon. The team reassessed donor safety, recipient risk, feasibility of a second transplant attempt, and strategies to reduce recurrent anaphylaxis risk. Intradermal testing in the recipient did not identify a definite trigger. During the same admission, she developed generalized urticaria that responded to corticosteroids and antihistamines, supporting the possibility of nonspecific environmental hypersensitivity consistent with her history. The second operation was scheduled 3 weeks after the postponed transplant. This interval was established to ensure complete clinical stabilization of both individuals and facilitate reassessment of the recipient allergy risk and donor wound healing. Additionally, it enabled informed consent and coordinated operative planning while minimizing delays that could further increase fibrosis and adhesions. The definitive strategy involved a 3‐day preoperative regimen of antiallergic medication for the recipient, followed by inducing recipient anesthesia first and observing the recipient for 15 min postinduction to ensure hemodynamic stability. The recipient iliac fossa was then prepared and proceeded with donor nephrectomy through the same flank incision.

### 3.6. Recipient Surgical Procedure During Reattempted Transplantation

The recipient was anesthetized first and positioned supine. After hemodynamic stability was confirmed, the right iliac fossa was approached through a 12‐cm Gibson incision. The peritoneum was mobilized medially, and the external iliac artery and vein were prepared for vascular anastomosis. The recipient remained stable during this preparation.

### 3.7. Redo Donor Nephrectomy via the Same Flank Incision

**Figure 2:**
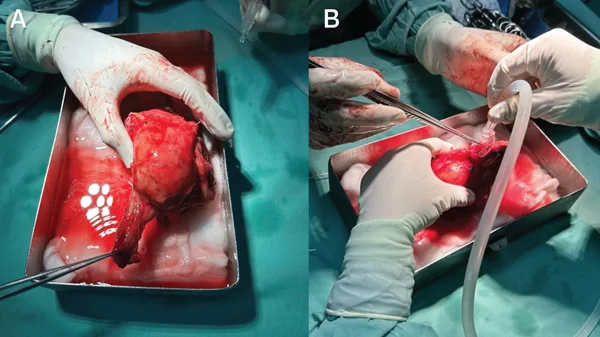
Preparation of the donor kidney during reattempted transplantation. (A) Retrieved donor kidney after redo nephrectomy through the original flank incision. (B) Graft perfusion and vascular inspection before implantation.

The donor was reapproached through the original left flank incision to avoid a second incision and preserve the retroperitoneal area used during the initial operation. Although dense adhesions were anticipated, the surgical team selected the same incision because the previous operation had already exposed the retroperitoneal space and renal hilum. Conversion to a new anterior or transperitoneal incision remains a backup solution if a safe dissection of the renal hilum cannot be achieved. The major intraoperative challenge was extensive retroperitoneal and perirenal adhesions involving the posterior kidney, lower pole, and ureter. These adhesions increased the risk of injury to the colon, pancreas, great vessels, and ureter. To improve orientation and avoid blind tension in the scarred retroperitoneum, the posterior peritoneum was opened. This technique allowed safer exposure of the renal hilum, aorta, ureter, and remaining renal surface. Conversion to a broader or alternative approach remained available if vascular control or organ safety became uncertain. However, exposure was adequate after posterior peritoneal opening, and no conversion was required. The renal artery, renal vein, and ureter measured approximately 3, 5, and 10 cm in length, respectively. After careful dissection of dense adhesions and hilar exposure, the renal vessels were secured using Hem‐o‐lok clips, and the kidney was successfully retrieved. The graft was subsequently flushed and washed with cold Custodiol solution and transferred to the recipient operating room for transplantation (Figure 2). The total donor operating time was approximately 3 h. Warm ischemia time was 2 min, cold ischemia time was 20 min, extraction time was 2 min, and estimated blood loss was 200 mL. The donor incision was then closed using the same layered closure technique as in the initial operation.

### 3.8. Transplantation

After graft arrival, the donor kidney was implanted extraperitoneally in the right iliac fossa through the prepared Gibson incision. The renal vein was anastomosed end‐to‐side to the external iliac vein, followed by end‐to‐side anastomosis of the renal artery to the external iliac artery using continuous 5‐0 polypropylene sutures. After vascular unclamping, the graft demonstrated immediate homogeneous reperfusion and prompt urine production. Ureteroneocystostomy was subsequently performed using the Lich–Gregoir technique over a 6‐Fr double‐J ureteral stent. A surgical drainage tube was placed, and the incision was closed in layers. The redo donor nephrectomy lasted almost 3 h, whereas vascular anastomosis and graft implantation in the recipient required an additional 40 min after graft arrival.

### 3.9. Follow‐Up and Outcomes

The postoperative recovery was uneventful for both donor and recipient, and no adverse or unanticipated postoperative complications were observed. The recipient showed appropriate urine output and allograft function normalized by postoperative Day 7. The donor was discharged on postoperative Day 7 in stable condition. Although no major postoperative complication occurred, the recipient remained hospitalized for 35 days. This prolonged admission was not related to graft dysfunction or any postoperative adverse complication. The recipient lived approximately 1000 km from the transplant center, making early return visits difficult and limiting access to specialized transplant follow‐up. According to our institutional protocol, the double‐J ureteral stent is routinely removed approximately 4 weeks after kidney transplantation. Therefore, the transplant team preferred to continue inpatient follow‐up until ureteral stent removal and until the maintenance immunosuppressive regimen had been appropriately adjusted and stabilized. The recipient was discharged after stent removal with stable graft function and a stable immunosuppressive regimen. The overall clinical progression is summarized in Table 2.

**Table 2:** Timeline of the interrupted and reattempted living donor kidney transplantation.

Time point	Clinical events
Day 0	Initial living donor nephrectomy and recipient transplantation were initiated
Day 0	Recipient developed intraoperative anaphylactic shock shortly after anesthesia induction
Day 0	Donor nephrectomy was discontinued before renal vessel ligation and kidney retrieval
Day 0	Donor and recipient were transferred to the intensive care unit for monitoring and stabilization
During hospitalization	Multidisciplinary reassessment and allergy evaluation were performed
3 weeks later	Reattempted transplantation was planned after recipient stabilization and renewed informed consent
Day of reattempted surgery	Recipient anesthesia and iliac vessel preparation were performed first to confirm hemodynamic stability
Day of reattempted surgery	Redo donor nephrectomy was successfully completed through the original flank incision
Postoperative Day 7	Donor discharged in stable condition
Postoperative Day 35	Recipient discharged in stable condition

## 4. Discussion

This case highlights three clinically notable features. Firstly, the transplantation was interrupted after exposure of the donor kidney but before completion of donor nephrectomy. Secondly, after comprehensive multidisciplinary reassessment and stable condition of the recipient, a decision was made to proceed with reattempted transplantation. Finally, the repeat donor nephrectomy was conducted through the original flank incision and required modification of the retroperitoneal dissection because of dense adhesions.

Given that the donor receives no medical benefits, living donor nephrectomy must be planned by the fundamental principle of minimizing donor risk. Accordingly, KDIGO guidelines and contemporary donor safety literature emphasize the importance of individualized donor evaluation, perioperative risk assessment, and careful long‐term follow‐up [2, 7]. In the present case, aborting the initial operation prevented implantation of the donor kidney into a hemodynamically unstable recipient and avoided unnecessary donor nephrectomy. This decision preserved both donor safety and graft viability; however, it subsequently created a more challenging secondary surgery.

Published evidence on redo living donor nephrectomy following an aborted transplant remains limited. Khan et al. recently reported surgical complexities in a redo donor–recipient transplant setting after an aborted transplant, highlighting that reattempted transplantation can be feasible but requires careful operative planning and anticipation of altered anatomy [4]. In contrast, our case differs because the donor kidney was reapproached through the same flank incision after prior open exposure, where the principal technical challenge arose from dense retroperitoneal adhesions around the kidney, lower pole, ureter, and hilar structures.

The decision to reapproach via the same flank incision was deliberate and strategically justified. Although laparoscopic or robot‐assisted donor nephrectomy may reduce incision‐related morbidity and support earlier postoperative recovery, their role in repeat donor nephrectomy remains unclear. In a redo procedure, previous retroperitoneal dissection may lead to early fibrosis, loss of normal tissue planes, and adhesions around the ureter and renal hilum. These changes may increase the risk of vascular, ureteral, or adjacent organ injury, particularly when exposure is limited. Therefore, the choice of surgical approach should be individualized according to the findings of the previous operation, the expected extent of adhesions, donor vascular anatomy, and the experience of the surgical team. Donor safety should remain the main consideration, and conversion to open surgery should be planned if adequate exposure or vascular control cannot be achieved. In the present case, re‐entry through the previous open flank incision was considered the safer option because it provided direct access and tactile control during dissection of the scarred renal hilum. However, a minimally invasive approach may be considered in carefully selected cases when adhesions are expected to be limited and the procedure is performed by surgeons experienced in both donor nephrectomy and complex reoperative renal surgery.

Adhesions are a well‐recognized contributor to operative complexity, especially in patients with prior abdominal or retroperitoneal intervals. In laparoscopic donor nephrectomy, perirenal adhesions have been associated with increased operative difficulties due to obscured tissue planes and complicated hilar dissection [8]. Similarly, in high‐risk donors with vascular anomalies and previous surgery history, careful preoperative planning and procedural flexibility were emphasized as crucial factors for safe nephrectomy [9]. Although these findings are not directly equivalent to a redo open donor nephrectomy through the same flank incision, they support the broader principle that adhesions do not contraindicate donor nephrectomy but increase the need for experienced surgical evaluation.

In our case, opening the posterior peritoneum was a pivotal intraoperative modification. Continued dissection with a scarred retroperitoneal space could enhance injury vulnerability of adjacent structures, including the colon, pancreas, ureter, or vessels. Posterior peritoneal entry improved anatomic orientation and facilitated safer exposure of the renal hilum and vascular landmarks. Importantly, this approach should not be considered a routine option but rather a case‐specific adaptation to restore operative clarity in a scarred retroperitoneum.

The recipient‐related complication emphasizes the importance of systematic evaluation following suspected anaphylaxis. Current anaphylaxis guidelines identify intramuscular epinephrine or adrenaline as first‐line therapy and advocate for subsequent allergist evaluation to identify preventable triggers [5, 6]. In this case, the inducer could not be definitively confirmed but negative intradermal testing, clinical stabilization, preoperative prophylaxis, and careful observation after induction enabled a controlled second attempt at transplantation. The inability to confirm a causative agent remains a limitation and should be acknowledged transparently.

Overall, although this report is limited by its single‐case design, comprehensive perioperative data including exact ischemia times, blood loss, fluid administration, and serial renal function values were provided. These variables strengthen interpretation of donor safety and allograft outcomes.

## 5. Conclusion

This case demonstrates that redoing open living donor nephrectomy through the original flank incision can be successfully performed after an aborted kidney transplantation in carefully selected individuals. Nevertheless, early postoperative adhesions and distorted retroperitoneal space may substantially enhance technical difficulty; the uniqueness of this case is expressed by the successful reapproach through the same incision following interrupted donor surgery caused by recipient intraoperative anaphylactic shock. Therefore, comprehensive multidisciplinary reassessment, coordinated perioperative plan, and intraoperative adaptability are essential to ensure donor safety and achieve favorable transplant outcomes.

## Author Contributions

Hung Ngoc Pham, Thang Vuong Hoang, and Hieu Van Le participated in surgical management and perioperative patient care. Phuc Dai Hong Vo, Anh Van Quoc Nguyen, and Tuan Minh Truong collected clinical data and contributed to manuscript preparation. Hung Ngoc Pham, Can Van Truong, and Tuan Kim Nguyen have supervised clinical management and critically reviewed the manuscript. John Edmondson, Kaitlyn Maddra, and Lance Hampton from Virginia Commonwealth University participated in academic discussion, manuscript drafting, critical revision, and interpretation of the surgical and transplant‐related aspects of the case through international academic collaboration.

## Funding

No funding was received for this manuscript.

## Disclosure

All authors reviewed and approved the final manuscript.

## Consent

Written informed consent for publication was obtained from both the donor and the recipient.

## Conflicts of Interest

The authors declare no conflicts of interest.

## Data Availability

The data supporting the findings of this case report are available from the corresponding author upon reasonable request.
